# Proteome changes in mesenteric lymph induced by sepsis

**DOI:** 10.3892/mmr.2014.2580

**Published:** 2014-09-18

**Authors:** PING ZHANG, YAN LI, LIAN-DONG ZHANG, LIANG-HUA WANG, XI WANG, CHAO HE, ZHAO-FEN LIN

**Affiliations:** 1 Emergency Department, Changzheng Hospital, Second Military Medical University, Shanghai 200003, P.R. China; 2 Emergency Department, Kunming General Hospital, Kunming, Yunnan 650032, P.R. China; 3 Emergency Department, Shanghai First People’s Hospital, Shanghai Jiao Tong University, Shanghai 201620, P.R. China; 4 Emergency Department, Shuguang Hospital Baoshan Branch, Shanghai University of Traditional Chinese Medicine, Shanghai 201900, P.R. China; 5 Department of Biochemistry and Molecular Biology, Second Military Medical University, Shanghai 200433, P.R. China

**Keywords:** lymph, proteome, sepsis, cecal ligation and puncture, bioinformation

## Abstract

The present study aimed to examine the changes in mesenteric lymph during the development of sepsis and to identify the distinct proteins involved, as targets for further study. The sepsis animal model was constructed by cecal ligation and puncture (CLP). The mesenteric lymph was collected from 28 adult male Sprague-Dawley rats, which were randomly divided into the following four groups (n=7 per group): CLP-6 h, CLP-24 h, sham-6 h and sham-24 h groups. Capillary high performance liquid chromatography-tandem mass spectrometry was performed to analyze the proteome in mesenteric lymph. A comprehensive bioinformatic analysis was then conducted to investigate the distinct proteins. Compared with the sham group, 158 distinct proteins were identified in the lymph samples from the CLP group. Five of these proteins associated with the same lipid metabolism pathway were selected, apolipoprotein E (ApoE), annexin A1 (Anxa1), neutrophil gelatinase-associated lipocalin (NGAL), S100a8 and S100a9. The expression of ApoE, Anxa1, NGAL, S100a8 and S100a9 were all elevated in the progression of sepsis. The five proteins were reported to be closely associated with disease development and may be a potential target for the diagnosis and treatment of sepsis. In conclusion, identifying proteome changes in mesenteric lymph provides a novel perspective to understand the pathological mechanisms underlying sepsis.

## Introduction

Sepsis, a systemic inflammatory response syndrome, is a leading cause of mortality in critically ill patients (1). Sepsis resulting from severe infection is frequently associated with confusion, metabolic acidosis, low blood pressure, decreased systemic vascular resistance and blood coagulation dysfunctions (2). Severe sepsis may lead to organ dysfunction and septic shock (3). Although the recommendations of the treatment targeting for sepsis are updated every four years (4–6), the mortality rate resulting from sepsis remains high, at between 30 and 50% (7).

To decrease the mortality rate and identify an effective treatment for sepsis, numerous studies have focused on identifying the pathogenic mechanisms that underlie sepsis. Disease-conditioned mesenteric lymph, but not portal blood, carries high levels of gut-derived factors leading to organ injury (8,9). As outlined in previous studies, protein fractions in the lymph are partly responsible for gut-origin sepsis (10,11). In addition, certain proteins in the lymph promote the progression of sepsis by acting as carriers of biologically active molecules or effector molecules. With advances in modern technology, proteomic analysis in lymph has provided a number of breakthroughs in examining the mechanisms underlying this critical disease (12–15).

Previously, investigations concerning sepsis have primarily focused on the proteome in the plasma (16,17), heart (1), liver (18) and skeletal muscle (19); however, there is little information regarding the role of lymph proteins. The experimental sepsis model induced by cecal ligation and puncture (CLP) has been widely used to reproduce numerous pathophysiological features of clinical sepsis (20,21). The significant changes in the levels of plasma proteins in mice with CLP-induced sepsis were reported to be during the late stage of septic development (24 h following surgery) (17). Therefore, in the present study, a sepsis model was constructed by CLP and the lymph was collected at 6 h and 24 h following surgery, which represented the early and the late stages of the acute phase, respectively. The aim was to examine the changes in rodent proteome mesenteric lymph in response to sepsis, in order to contribute to the current understanding of the pathogenesis of sepsis progression.

## Materials and methods

### Animals

A total of 28 adult male Sprague-Dawley rats (Slaccas Laboratory Inc., Shanghai, China), weighing 250–300 g, were housed at 25°C and subjected to 12 h light/dark cycle treatment conditions. All of the animals were fed a standard rodent diet (Slaccas Laboratory Inc.) with access to water *ad libitum*. All of the animals received humane care in accordance with the recommendations of the Guide for the Care and Use of Laboratory Animals. Approval was obtained from the Institutional Animal Care and Use Committee at the Second Military Medical University (Shanghai, China).

### Experimental model

The animals were randomly divided into the following four groups (n=7 per group): CLP-6 h, sham-6 h, CLP-24 h and sham-24 h groups. In the CLP groups, the sepsis models were constructed by CLP as previously described (22). Briefly, general anesthetic was induced by intraperitoneal injection of pentobarbital (40 mg/kg; Sinopharm Chemical Reagent Co., Ltd., Shanghai, China) and CLP was performed with a single in-and-out puncture (20 gage needle; Yangzhou Great Wall Medical Equipment Factory, Yangzhou, China) at the middle part of the cecum. To ensure successful puncture, droplets of feces were extruded from the penetration holes. The rats in the sham groups underwent cecum exenteration under general anesthetic instead of CLP. After surgery, all the rats were injected with 100 g/5 ml of normal saline subcutaneously. Following recovery from the anesthesia, the rats received free access to food and water *ad libitum*.

### Lymph collection

Prior to the CLP/sham surgery, the thoracic duct was cannulated with micro-urethane tubing (inner diameter, 0.635 mm; outer diameter, 1.02 mm; American Health & Medical Supply International Corp. Co., Ltd., New York, NY, USA) and externalized through a 14-gage angiocatheter (Hospira, Inc., Lake Forest, IL, USA) in the left flank of all the experimental animals. At 6 h and 24 h following CLP/sham surgery, the lymph samples were collected from all groups. Lymph collection was conducted by thoracic duct drainage according to a modified method that has been previously described (23). In brief, the rats were re-anesthetized with intraperitoneal injection of 40 mg/kg pentobarbital. The lymph samples were collected directly into sterile ice-chilled vacuum EDTA tubes (Shanghai Kehua Bio-engineering Co., Ltd., Shanghai; China) for 60 min. One sample from the seven rats within one group was centrifuged at 4°C (1,700 × g) for 15 min and immediately stored at −80°C for further analysis.

### Proteomic analysis

Proteomic analysis, according to a previous description (24), and bioinformatic analysis were performed by Sensichip Infotech Co., Ltd. (Shanghai, China). The bioinformatics analysis was used to analyze the molecular functions of differentially expressed genes of the identified proteins. All of the chemical reagents, software and equipment were provided by this company. The experimental procedures were performed according to standardized manufacturer’s instructions.

### Major protein depletion

The lymph samples were defrosted, diluted and filtered through a 0.22-μm filter membrane (AB SCIEX, Framingham, MA, USA). Next, three major abundant proteins (albumin, IgG and transferrin) were depleted by the Multiple Affinity Removal Column (M-3; Agilent Technologies Co., Ltd., Santa Clara, CA, USA). When the samples had been individually depleted, quantitative analysis of the lymph samples was performed with the *Dc* protein assay reagent (Bio-Rad, Hercules, CA, USA). The samples were then desalted and concentrated by ultrafiltration with a Vivaspin 4 centrifugal concentrator and 5-kDa polyethersulfone filter (Sartorius AG, Göttingen, Germany). The target proteins were detected by 12.5% sodium dodecyl sulfate-polyacrylamide gel (SDS-PAGE) electrophoresis.

### Capillary high performance liquid chromatography-tandem mass spectrometry (HPLC-MS/MS)-based proteomics

The proteins from each sample (150 μg) were dissolved in 200 μl UA buffer (8 M urea in 150 mM Tris-HCl; pH 8.0). The solution was centrifuged (14,000 × g, 30 min) twice and the supernate was abandoned. The protein sample was then incubated with 100 μl iodoacetamide in the dark at room temperature for 30 min. Following centrifugation (14,000 × g, 20 min), the sample was mixed with 100 μl UA buffer followed by reduplicate centrifugation (14,000 × g, 20 min). The protein sample underwent reduction (blending with 100 μl dissolution buffer and reduplicate centrifugation at 14000 × g for 20 min) and was digested in 40 μl trypsin buffer (2 μg trypsin in 40 μl dissolution buffer) at 37°C for 16–18 h. Following centrifugation (14,000 × g, 10 min) in a new collection tube, the peptides were then eluted and quantified at optical density (OD)_280_ in the Unicam SP 600 spectrophotometer (Pye Unicam Ltd., Cambridge, UK)

The sample (50 μg) was labeled with iTRAQ reagents (iTRAQ Reagent-4 plex Multiplex kit; AB SCIEX) according to the manufacturer’s instructions. Samples from CLP-6 h, CLP-24 h, sham-6 h and sham-24 h groups were labeled with 114, 115, 116 and 117 iTRAQ isobaric tags, respectively. The labeled materials were then combined and pre-fractionated on a polysulfoethyl 4.6×100-mm column (5 μm, 200 Å; PolyLC Inc., Columbia, MD, USA) using an AKTA Purifier 100 (GE Healthcare, Little Chalfont, UK). Approximately 30 effluent fractions were collected and merged into ten fractions according to the strong cation exchange chromatogram. The fractions were freeze-dried and desalted using a C_18_ cartridge (Sigma, St. Louis, MO, USA).

Subsequently, HPLC analysis was performed. The samples were added to the sample column (2 cm × 100 μm, 5 μm-C18; EASY-Column Capillary Column; AB Sciex, Waltham, MA, USA) automatically and were then separated through an analytical column (75 μm × 100 mm, 3 μm-C18; EASY-Column Capillary Column; Thermo Fisher Scientific). The HPLC Easy nLC gradient between buffer A (0.1% formic acid in water) and buffer B (0.1% formic acid in acetonitrile) was formed at 250 μl/min as follows: Increasing to 35% B from 0–100 min, increasing to 100% B by 108 min and held at 100% until 120 min. The sample effluent was directed into the ion spray source of a Q-Exactive mass spectrometer (Thermo Finnigan, San Jose, CA, USA) scanning from 300 to 1800 m/z for 120 min.

### Sequence database searches

The sequence database searches and quantitative analysis were performed by Mascott 2.2 software (Matrix Science Ltd., Boston, MA, USA), Proteome Discoverer 1.3 and Xcalibur softwares (Thermo Fisher Scientific).

The data were searched against the ipi.RAT.v3.87.fasta database (sequence no. 39925) using Mascot 2.2 software with the search parameters as follows: Type of search, MS/MS ion search; enzyme, trypsin; mass values, monoisotopic; max missed cleavages, 2; fixed modifications, carbamidomethyl (C), iTRAQ4plex (N-term), iTRAQ4plex (K); variable modifications, oxidation (M), iTRAQ4plex (Y); peptide mass tolerance, ±20 ppm; and fragment mass tolerance, 0.1 Da. Peptide false discovery rates ≤0.01 and a peptide Mascot score >20 were defined as significant.

### Data analysis

Quantitative analysis was conducted using Proteome Discoverer 1.3 software according to the peak intensity of labeled peptides. For different samples labeled with different tags, the peptide quantification in one sample was calculated as the ratio of the signal intensity of one tag compared with other the tags. The median of the calculated ratios was considered as the result of protein quantitation. Each median of the signal ratio was normalized to eliminate human sample loading error. Differences in the relative abundance were calculated as differences in the log peak areas and reported as fold changes between two groups. Differences among the groups were analyzed by Student’s t-test. P<0.05 was considered to indicate a statistically significant difference. Differentially expressed proteins were screened by the t-test (P<0.05) and fold change (fold change>1.5) method.

### Bioinformatics

In the gene ontology (GO) analysis, differentially expressed genes (DEGs) encoding the 158 identified proteins were mapped to the GO database using the GSEABase software package on the R statistics platform (http://www.r-project.org/). DEGs were classified according to three ontologies: Biological processes, cellular components and molecular functions.

In the pathway analysis, DEGs were mapped to the Kyoto Encyclopedia of Genes and Genomes (KEGG) database (www.bioconductor.org) by GenMAPP v2.1 (Gladstone Institutes, San Francisco, CA, USA) and the enrichment degree of each gene was counted in different pathways.

In the network analysis, the interactions between DEGs were analyzed by downloading the pathway data from the KEGG, MIPS (http://mips.helmholtz-muenchen.de/proj/ppi/) and PubMed databases (25) and KEGGSOAP software package (www.bioconductor.org). Inter-correlations between DEGs were analyzed using the co-citation algorithm.

Finally, the results of the three types of analyses were integrated into a network of protein-protein correlations with comprehensive consideration. The network was illustrated graphically using Medusa software (Informer Technologies, Inc., Bilbao, Spain).

## Results

### Sepsis model

Following induction of the sepsis model, the rats in the sham and CLP groups presented physiological changes of different degrees.

At 6 h following surgery, the rats in the sham-6 h group presented with normal physical ability. The mental state (assessed by observation of movements of the animal) and weight loss of rats was marginally lower than prior to surgery. The rats were responsive, and actively feeding and drinking. No piloerection or abdominal distension were observed and the rats had dry granular feces, soft stools and a clean anus. At 24 h, the rats in the sham-24 h presented with a similar physiological status.

However, rats in the CLP-6h group exhibited poor mental state, decreased coat glossiness, piloerection, abdominal distension, and a fairly sensitive response to stimuli (stimuloation by a needle in the tail). The rats stayed in groups, did not eat and only about half of them were actively drinking water. The abdominal incisions of the rats were red with a small quantity of exudate and stools were soft or loose. In the CLP-24 h group, the rats were more sluggish and also stayed in groups. The rats had evident abdominal distention, decreased eating and drinking habits and secretion from their eyes. The rats responded slowly in spite of intense stimulation. Feces were observed on the anuses and tails of the rats. The perianal hairs of the rats were wet and the feces were primarily loose stools.

The mortality rate was 60% in the CLP groups following ten days post-operation.

### Sample preparation

After the highly-abundant protein was depleted, the concentrations of the remaining lymph protein in the CLP-6, CLP-24, sham-6 and sham-24 groups were 1.52, 1.54, 1.51 and 2.09 μg/μl, respectively. By quantitative analysis, the relative concentrations of peptides were estimated by absorbance at 280 nm (OD_280_1.1=1 μg/μl). The OD_280_ of the samples were 1.31 (CLP-6), 1.63 (CLP-24), 1.44 (sham-6) and 1.39 (sham-24), respectively. The depletion rate of the highly-abundant protein was relatively high, while the concentration of the target proteins was low.

### Lymph proteomics

The abundances of 984 proteins identified in the rat lymph samples were compared between the CLP-6 and sham-6; CLP-24 and sham-24; CLP-6 and CLP-24; and sham-6 and sham-24 groups. A total of 158 distinct proteins with a fold change ≥1.5; P<0.05 between the different groups were identified in the present study. Compared with the sham-6 h group, 30 of the 158 proteins were significantly increased in the CLP-6 h group (Table I). These proteins were listed according to their molecular function (using the freely available protein database UniProt; http://www.uniprot.org/) and there were shown to be 13 binding proteins, two structural proteins, three enzymes, two protease inhibitors, one cytokine, one transcription factor, five uncharacterized proteins and three proteins with unclear function. An additional uncharacterized protein, Pfkp (fold=0.28, P=0.0016), was found to decrease significantly (fold change≤0.3, P<0.05) in the CLP-6 group.

Compared with the sham-24 group, 20 proteins were confirmed to increase significantly (fold change≥1.5; P<0.05) in abundance of the CLP-24 group (Table II) and included six binding proteins, two enzymes, one protease inhibitor, one signal transducer protein and ten uncharacterized proteins. By contrast, two proteins were identified to decrease significantly (fold change≤0.3; P<0.05) in the CLP-24h group compared with the sham-24 group: Svs4 (fold=0.25, P=0.0016) and one uncharacterized protein, Svs6 (fold=0.27; P=0.0016).

### Bioinformatics

**Figure 1:**
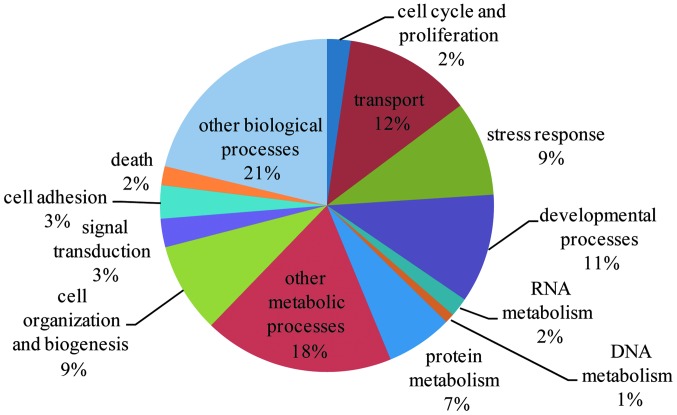
Biological process analysis. The chart shows the biological processes that the 158 proteins are involved in.

**Figure 2:**
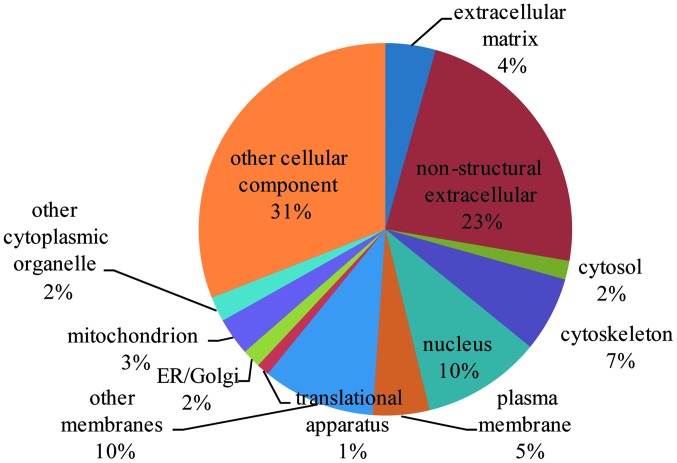
Cellular component analysis. The chart shows the cellular components that the 158 proteins are involved in.

**Figure 3:**
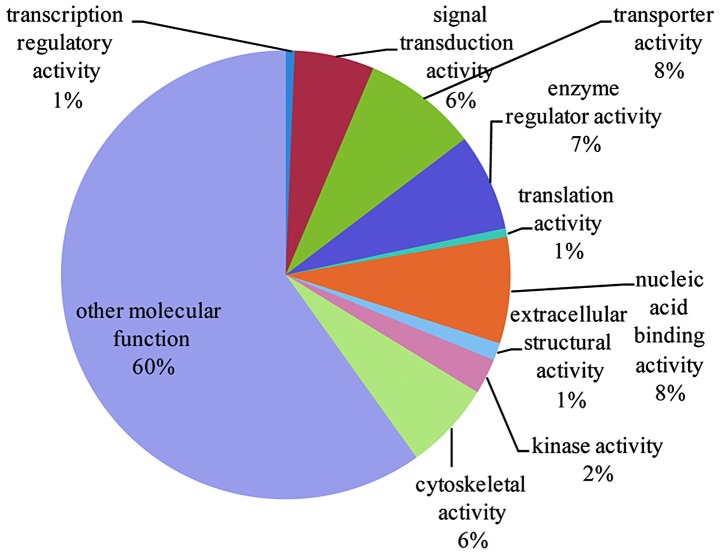
Molecular function analysis. The chart shows the function the 158 proteins are involved in.

The 158 proteins identified in the CLP and sham groups were further analyzed for functional and biological relevance. Using GO analysis, the proteins were analyzed based of their involvement in biological processes, cellular components and molecular functions (Figs. 1–3 and cross-referenced to Tables III–V).

Following pathway analysis, the 158 proteins were shown to be mainly involved in extracellular matrix-receptor interactions (count=7; P<0.01), focal adhesions (count=7; P<0.05), and complement and coagulation cascades (count=6; P<0.01; Table VI).

**Figure 4:**
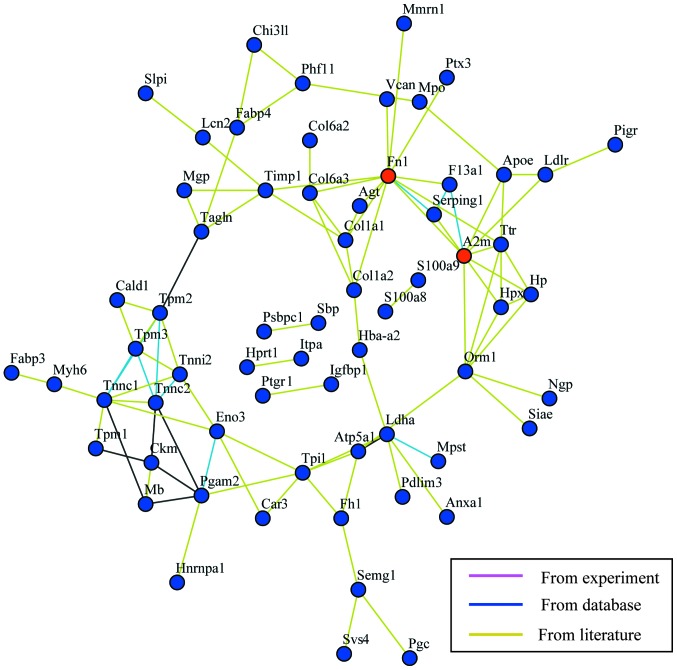
Differentially expressed protein network analysis (1), using gene symbols. The protein-protein interaction network of differentially expressed proteins; the gene with the highest connectivity in network connectivity analysis are termed the hub genes, e.g. Fn1 and A2m. Red, hub gene; blue, general gene.

**Figure 5:**
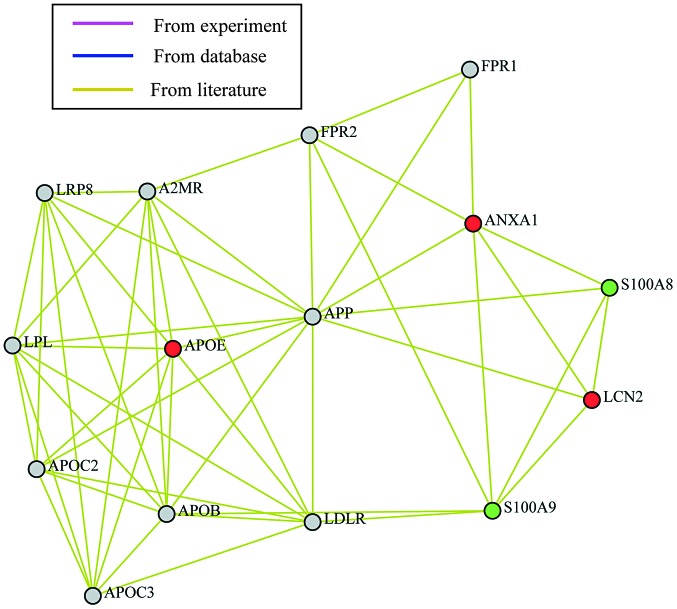
Differentially expressed protein network analysis (2), using gene symbols. It is observed that the five selected proteins: ApoE, Anxa1, NGAL (Lcn2), S100a8 and S100a9 are involved in the same lipid metabolism-associated network, with amyloidogenic glycoprotein (APP) as the hub. ApoE, apolipoprotein E; Anxa1, annexin A1; NGAL, neutrophil gelatinase-associated lipocalin. Red, upregulated genes (CLP24 vs. CLP-6); green, downregulated genes (CLP-24 vs. CLP-6).

**Figure 6:**
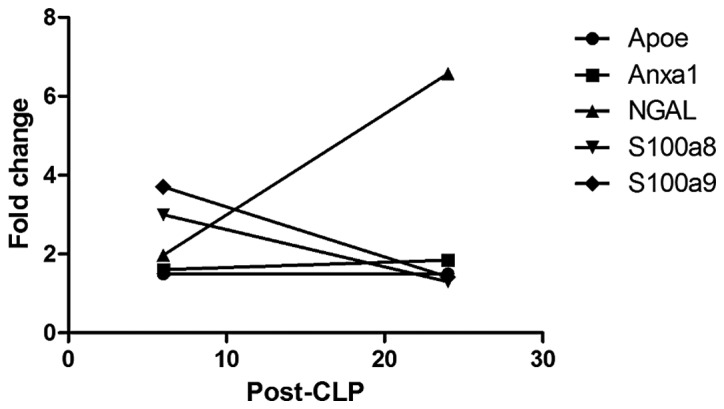
Relative abundance of five selected proteins at 6 h and 24 h following CLP. Of the five selected proteins, the relative abundance of S100a9 exhibited the greatest fold change 6 h following CLP (fold change, 3.7; P<0.0001), followed by S100a8 (fold change, 3; P<0.0001). The relative abundance of ApoE and Anxa1 were almost unchanged from 6 to 24 h following CLP. The levels of S100a8/S100a9 progressively decreased, while NGAL increased rapidly. ApoE, apolipoprotein E; Anxa1, annexin A1; NGAL, neutrophil gelatinase-associated lipocalin.

The gene symbols were mapped on a protein-protein interaction network (Fig. 4). The closest connection was identified between hub Fn1 and A2m genes. However, no significant fold-change was found in the Fn1 gene and A2m-gene associated protein expression. This network only shows the peripheral interactions between the differentially expressed proteins, but not the close interactions between individual proteins. Therefore, further analysis of the time-course data was performed. Comprehensively considering the fold change (>1.5 or <0.5) and a P-value <0.05, the biological effects of the distinct proteins and involvement of proteins in the induction of inflammation and sepsis, five proteins were selected for further gene network analysis, including apolipoprotein E (ApoE), annexin A1 (Anxa1), neutrophil gelatinase-associated lipocalin (NGAL), S100a8 and S100a9 (Fig. 5). Compared with the sham-6 h group, Anxa1 and ApoE were upregulated in the CLP-6 h group. There was no significant difference in the expression of Anxa1 and ApoE between the CLP-24 h and CLP-6 h groups. S100a8 and S100a9 were overexpressed at 6 h following CLP compared with the sham-6 h group, while downregulated 24 h after CLP. In addition, the expression of NGAL was detected to be elevated in the CLP-6 h (compared with sham-6 h) and CLP-24 h groups (compared with CLP-6 h group). All of the five proteins were found to be involved in the same lipid metabolism-associated network, with amyloidogenic glycoprotein as the hub. The alterations in the relative abundance of these five proteins between the CLP-6 h and CLP-24 h groups are demonstrated in Fig. 6. Among the proteins, NGAL expression revealed a significant increase from CLP-6 h to CLP-24 h.

## Discussion

Although numerous investigations concerning sepsis have been conducted, the detailed and precise mechanism of sepsis remains unclear. The present study provided a novel prospective to aid in the elucidation of the mechanism of sepsis by determining proteome changes in the mesenteric lymph. The results demonstrated that a total of 158 proteins in the mesenteric lymph, which were found to exist in all of the groups, were identified to be significantly differentially expressed (fold change ≥1.5; P<0.05). The 158 proteins demonstrated abundant differences between the CLP and sham groups as well as the CLP-6 h and CLP-24 h groups. Following analysis of the 158 proteins by bioinformatic methods, five proteins were investigated as targets: ApoE, Anxa1, NGAL, S100a8 and S100a9.

ApoE, the ligand of the low density lipoprotein receptor, has a vital role in lipid metabolism and is involved in the pathogenesis of critical diseases (26,27). It has been suggested that ApoE polymorphisms are associated with severe sepsis in surgical patients (28). In a previous study, the majority of patients with severe sepsis did not have the *APO*ɛ*3* allele. The *APO*ɛ*3* allele significantly protected carriers against the risk of severe sepsis (29). Although the role of ApoE in sepsis was not clear, ApoE was determined to be associated with a variety of different factors. In sepsis models induced by CLP, ApoE reversed the increase of hepatic T cell apoptosis and necrosis, and promoted Th1 cytokine levels (30). In addition, ApoE^−/−^ mice demonstrated notably higher levels of proinflammatory cytokines in the serum, compared with wild-type mice (31,32). ApoE inhibited the lipopolysaccharide (LPS)-induced increase of tumor necrosis factor-α, interleukin (IL)-1β and IL-6 in serum and decreased the mortality rates of mice with sepsis (32). By contrast, another study suggested that ApoE increased the mortality rates of rats with sepsis (30). Seven days following CLP, rats injected with recombinant ApoE3 had a mortality rate of 82.14 and 100% at doses of 114 μg/kg or 1.6 mg/kg, respectively, while none of the saline-injected rats died (30). The results demonstrated that ApoE was involved in multiple biological processes, including transport, stress responses, cell organization and biogenesis. ApoE was also involved in transporter and enzyme regulator activity. At 6 h following CLP, ApoE was indicated to be significantly upregulated in the lymph, but not in the CLP-24 h group. Therefore, it is suggested that ApoE demonstrated protective effects in the early stages of sepsis, but that this effect was reversed in the later stages.

Anxa1, an annexin, is known to be an endogenous anti-inflammatory mediator (33). The expression of annexins (AnxA1–AnxA7, AnxA9 and AnxA11) has been demonstrated to be regulated by pro- and anti-inflammatory stimulation (34). Furthermore, pro- and anti-inflammatory responses were revealed to be present throughout the entire process of sepsis progression (35). Anxa1 decreased the degree of the acute inflammatory response (36) and exerted active effects on the suppression of acute inflammation by preventing the adhesion and migration of neutrophils, adjusting the production of pro-inflammatory and anti-inflammatory cytokines and promoting neutrophil apoptosis (37,38). There was evidence that endogenous Anxa1 exhibited a protective role in the cerebral microcirculation of sepsis (39). However, it was reported that the Anxa1 plasma level decreased in patients with sepsis, as compared with control patients (35). In the present study, Anxa1 was overexpressed in the lymph of the CLP-6 h group (fold=1.61, P=0.0103) compared with that in the sham-6 h group. It indicated that Anxa1 had a key role in sepsis progression and that the expression of Anxa1 may be different in the lymph and plasma of patients. Bioinformatics analysis revealed that Anxa1 was mainly involved in enzyme regulator activity, signal transduction and cell adhesion, which corresponded with previous studies.

S100a8 and S100a9 as members of the S100 protein family, are released from neutrophils and activate phagocytes during sepsis (40). S100a8 and S100a9 most commonly emerge as heterodimers and have a prominent role in the pathogenesis of various inflammatory diseases (41).

S100A8 and/or S100A9 have been reported to possess multiple important biological activities, including mediating neutrophil adhesion to fibronectin (42), inducing neutrophil chemotaxis (43) and mediating apoptosis (44). It has also been demonstrated that S100A8 and S100A9 enhanced inflammatory responses by mediating nuclear factor-κB activation and inducing cytokine secretion, including IL-6, IL-8 and IL-1β (41). During the early phase of sepsis, excessive release of proinflammatory cytokines and chemokines reflect hyperreactive immune responses and result in hyporeactive immune responses in the later phase along with intractable shock, refractory infection, multiple organ failure and even mortality (45). As outlined in a previous study, the S100a8/S100a9 plasma levels were significantly elevated in patients with severe sepsis (46). In abdominal sepsis patients, the S100a8/S100a9 levels in the abdominal fluid were >15-fold that in the plasma, indicating that S100a8/S100a9 expression was induced primarily at the site of infection during sepsis (46). The present study indicated that the expression of S100a8 and S100a9 were significantly elevated in the lymph during early stages of sepsis (CLP-6h) but decreased in later stages (CLP-24h). As S100 proteins have been defined as clinical markers of inflammation in a previous study (47), S100a8 and S100a9 levels in the lymph may be a marker for the diagnosis of early stage sepsis.

NGAL, a lipocalin, is overexpressed in acute kidney injury due to ischemia, toxic factors and sepsis (48,49). In patients with sepsis, the overexpression of NGAL attributed to the response of the kidneys to sepsis. NGAL expression was found to be elevated in the serum of patients with sepsis (50). The concentration of urinary NGAL was also considered to be an indicator of early acute kidney injury in patients with sepsis (49). Furthermore, NGAL levels in the urine were reported to be correlated with the plasma or serum levels (51). In the present study, NGAL levels in the lymph increased progressively and reached a peak at 24 h following CLP. The NGAL levels in the plasma and urine have been widely adopted as an early predictor of acute kidney injury (52–54). NGAL had a critical role in sepsis progression and further studies are required to determine its clinical utility as a diagnostic marker for sepsis.

In conclusion, the present study demonstrated that the expression of five proteins (ApoE, Anxa1, NGAL, protein S100a8 and S100a9) was significantly elevated in the progression of sepsis. All five proteins appeared to have vital roles in critical disease development and may therefore be potential targets for the treatment and diagnosis of sepsis. The present study provides a novel perspective to aid in the understanding of the pathological mechanism of sepsis.

## Figures and Tables

**Table I:** Upregulated proteins in the lymph of the cecal ligation and puncture-6 h group.

Gene name	Description	Fold change	P-value
Sbp	Uncharacterized protein	4.29	0.0000
Scgb2a1	Secretoglobin family 2A member 2	4.19	0.0000
S100a9	S100-A9 protein	3.73	0.0000
Ngp	Uncharacterized protein	3.37	0.0000
S100a8	S100-A8 protein	3.04	0.0000
Retnlg	Resistin-like molecule gamma	2.83	0.0000
Psbpc2	Prostatic steroid-binding protein C2	2.71	0.0000
Psbpc1	Prostatic steroid-binding protein C1	2.51	0.0000
Klk1c9	Submandibular glandular kallikrein-9	2.48	0.0000
Tceb2	Transcription elongation factor B polypeptide 2	2.38	0.0000
LOC298109	Uncharacterized protein	2.07	0.0000
Lmnb1	Lamin-B1	2.06	0.0000
Igfbp1	Insulin-like growth factor-binding protein 1	2.00	0.0002
Lcn2	Neutrophil gelatinase-associated lipocalin	1.97	0.0002
Fh1	Isoform Mitochondrial of Fumarate hydratase, mitochondrial	1.96	0.0002
Pigr	Polymeric immunoglobulin receptor	1.90	0.0002
Clca1	Uncharacterized protein	1.90	0.0002
Tsku	Tsukushin	1.89	0.0002
LOC679994	Histone H3.1	1.80	0.0004
	GF20391-like isoform 1	1.76	0.0010
Mpst	3-Mercaptopyruvate sulfurtransferase	1.71	0.0041
Andpro	Cystatin-related protein 1	1.69	0.0056
Myh6	Myosin-6	1.64	0.0074
Mmrn1	Uncharacterized protein	1.63	0.0072
Anxa1	Annexin A1	1.61	0.0103
Nono	Non-POU domain-containing octamer-binding protein	1.59	0.0123
ApoE	Apolipoprotein E	1.53	0.0265
Serpinb1a	Leukocyte elastase inhibitor A	1.52	0.0301
Chi3l1	Chitinase-3-like protein 1	1.51	0.0310
Ccdc48	EF-hand domain-containing protein ENSP00000381169 homolog	1.50	0.0311

**Table II:** Upregulated proteins in the lymph of the cecal ligation and puncture-24 h group.

Gene name	Description	Fold change	P-value
Siae	Sialate O-acetylesterase	3.60	0.0000
Lcn2	Neutrophil gelatinase-associated lipocalin	2.30	0.0000
Mgp	Matrix Gla protein	2.05	0.0001
Ldlr	Uncharacterized protein	1.81	0.0004
Ngp	Uncharacterized protein	1.81	0.0004
RGD1565682	Uncharacterized protein	1.81	0.0004
Ptgr1	Prostaglandin reductase 1	1.80	0.0004
Sbp	Uncharacterized protein	1.78	0.0014
Tnnc1	Cardiac troponin C	1.70	0.0037
LOC299282	Uncharacterized protein	1.70	0.0033
Vcan	Uncharacterized protein (Fragment)	1.67	0.0057
Myh6	Myosin-6	1.64	0.0075
Sema6d	Uncharacterized protein	1.64	0.0074
Il1r2	Interleukin-1 receptor type 2	1.62	0.0102
Andpro	Cystatin-related protein 1	1.60	0.0114
Slc4a1	Uncharacterized protein	1.57	0.0148
Fgl2	Fibrinogen-like 2	1.56	0.0190
Mpo	Uncharacterized protein	1.54	0.0218
Chi3l1	Chitinase-3-like protein 1	1.52	0.0276
Itpa	Uncharacterized protein	1.51	0.0296

**Table III:** Biological process analysis.

Term	Gene count	P-value	Gene list
Cell cycle and proliferation	5	0.912015	Agt, Rcc2, Anxa1, Hprt1, Ak1
Transport	27	0.002065	ApoE, Hnrnpa1, Slc4a1, Agt, Fabp5, Anxa1, Ptx3, Ttr, Apof, Ldlr, Hpx, Fabp4, Mpst, Abp1, Fabp3, Lcn2, Clca1, Hba-a2, Fabp6, Myh6, Lbp, Tnnc1, Col1a1, Mb, Atp7a, Orm1, Atp5a1
Stress response	20	3.25E-05	F13a1, ApoE, Saa4, Thbs1, Agt, Ngp, Kng1, Ddb1, Nono, Mpo, Hpx, Fabp4, Fn1, Hspa1l, Serpina3n, Lbp, Hspa5, Serping1, Mb, Orm1
Developmental processes	23	0.069737	ApoE, Neb, Thbs1, Agt, Hist1h1b, Vcan, Mgp, Acan, Timp1, Hspa1l, Hba-a2, Myh6, Sema6d, Tpm1, Tpi1, Tagln, Hprt1, Pdlim3, Col1a1, Mb, Atp7a, Hexb, Atp5a1
RNA metabolism	4	0.999991	Hnrnpa1, Nono, Fabp4, Tceb2
DNA metabolism	2	0.74583	Ddb1, Nono
Protein metabolism	14	0.86033	F13a1, Pgc, Dpm1, Ddb1, Serpinb1a, Acy1, Hpx, Rps28, Tceb2, Serping1, Hp, Rpl8, Atp7a, sHexb
Other metabolic processes	40	2.44E-07	Aox1, ApoE, Agt, Fabp5, Dpm1, Ptx3, Acy1, Pgm1, Ttr, Apof, Car3, Ptgr1, Ldlr, Acan, Chi3l1, Car1, Mpo, Hpx, Fabp4, Abp1, Fabp3, Pfkp, Fabp6, Fh1, Pgam2, Lbp, Tpi1, Pgls, Pla1a, Ldha, Ckm, Hprt1, Ak1, Itpa, Eno3, Gstm1, Atp7a, Hexb, S100a9, Atp5a1
Cell organization and biogenesis	19	0.034714	ApoE, Neb, Agt, Hist1h1b, Rcc2, Hist1h1d, Ptx3, Ldlr, Acan, Fn1, Myh6, Lbp, Hprt1, Hist1h1a, Pdlim3, Atp7a, Hexb, S100a9, Hist1h2ai
Signal transduction	6	1	Fgl2, Agt, Anxa1, Fgl1, Hpx, Hspa5
Cell adhesion	7	0.103657	Thbs1, Agt, Vcan, Acan, Fn1, Col1a1, Col6a2
Death	4	0.788118	Fgl2, Agt, Hprt1, Atp7a
Other biological processes	46	0.332232	ApoE, Pgc, Mrfap1, Agt, Ngp, Kng1, Rcc2, Sbp, Gemin4, Ptx3, A2m, Mgp, Apof, Tpm2, Ldlr, Glrx3, Hpx, Fabp4, Igfbp1, Hspa1l, Retnlg, Serpina3n, Lcn2, Cald1, Fh1, Myh6, Sema6d, Tpm1, Lbp, Ldha, Myom2, Hprt1, Hist1h1a, Serping1, Tnnc1, Semg1, Col1a1, Rpl8, Mb, Atp7a, Orm1, S100a8, Hexb, S100a9, Mybpc1, Prg4

**Table IV:** Cellular component analysis.

Term	Gene count	P-value	Gene list
Extracellular matrix	8	0.00073	Vcan, Acan, Timp1, Fn1, Col1a2, Chad, Col1a1, Col6a2
Non-structural extracellular	43	0	F13a1, ApoE, Fgl2, Pgc, Saa4, Thbs1, Agt, Svs6, Kng1, Vcan, Svs4, Col6a3, Sbp, Ptx3, A2m, Mgp, Ttr, Apof, Ldlr, Acan, Chi3l1, Fgl1, Hpx, Timp1, Fn1, Abp1, Igfbp1, Col1a2, Serpina3n, Lcn2, Chad, Lbp, Pla1a, Pigr, Tsku, Serping1, Mmrn1, Hp, Col1a1, Orm1, Slpi, Col6a2, Prg4
Cytosol	3	0.430499	Pfkp, Ldha, Eno3
Cytoskeleton	12	0.028496	Neb, Slc4a1, Lmnb1, Rcc2, Tpm2, Tpm3, Cald1, Myh6, Tpm1, Myom2, Pdlim3, Mybpc1
Nucleus	19	0.988576	Hnrnpa1, Mrfap1, Lmnb1, Hist1h1b, Rcc2, Dpm1, Anxa1, Ddb1, Serbp1, Gemin4, Tsn, Hist1h1d, Nono, Fabp4, Hmgn2, Tceb2, S100a11, Hist1h1a, Hist1h2ai
Plasma membrane	9	0.99651	Slc4a1, Col6a3, Anxa1, Fn1, Clca1, Pigr, Ak1, Atp7a, Col6a2
Other membranes	18	1	Slc4a1, Lmnb1, Dpm1, Col6a3, Anxa1, Ldlr, Mpst, Fn1, Clca1, Cald1, Sema6d, Pigr, Il1r2, Ak1, Atp7a, Hexb, Col6a2, Atp5a1
Translational apparatus	2	0.39956	Rps28, Rpl8
ER/Golgi	3	0.993138	Dpm1, Hspa5, Atp7a
Mitochondrion	6	0.652947	Mpo, Mpst, Fh1, Ckb, Ak1, Atp5a1
Other cytoplasmic organelle	4	0.38439	Siae, Ldlr, Mpo, Hexb
Other cellular component	57	0.025085	Aox1, F13a1, Hnrnpa1, Mrfap1, Ngp, Fabp5, Hist1h1b, Rcc2, Dpm1, Siae, Anxa1, Ddb1, Serbp1, Serpinb1a, Tsn, Hist1h1d, Acy1, Apof, Tpm2, Car3, Ptgr1, Glrx3, Tpm3, Car1, Fabp4, Mpst, Hmgn2, Fabp3, Retnlg, Rps28, Pfkp, S100a11, Ostf1, Fabp6, Fh1, Myh6, Sema6d, Tpm1, Trim72, Tagln, Hspa5, Ldha, Ckm, Hprt1, Hist1h1a, Ckb, Pdlim3, Ak1, Itpa, Eno3, Semg1, Gstm1, Col1a1, Rpl8, Atp7a, Hexb, Hist1h2ai

**Table V:** Molecular function analysis.

Term	Gene count	P-value	Gene list
Transcription regulatory activity	1	0.999674	Fabp4
Signal transduction activity	9	0.999986	Fgl2, Agt, Ttr, Ldlr, Fgl1, Lbp, Pigr, Il1r2, Prg4
Transporter activity	13	0.030194	ApoE, Slc4a1, Fabp5, Fabp4, Fabp3, Lcn2, Clca1, Hba-a2, Fabp6, Mb, Atp7a, Orm1, Atp5a1
Enzyme regulator activity	11	0.006352	ApoE, Agt, Kng1, Anxa1, Serpinb1a, A2m, Timp1, Fn1, Serpina3n, Serping1, Slpi
Translation activity	1	0.285497	Hspa5
Nucleic acid binding activity	12	0.962945	Hnrnpa1, Hist1h1b, Dpm1, Ddb1, Serbp1, Tsn, Hist1h1d, Nono, Hmgn2, Hist1h1a, Rpl8, Hist1h2ai
Extracellular structural activity	2	0.013584	Col1a2, Col1a1
Kinase activity	4	0.84497	Pfkp, Ckm, Ckb, Ak1
Cytoskeletal activity	10	0.002977	Tpm2, Tnni2, Tpm3, Cald1, Myh6, Tpm1, Myom2, Pdlim3, Tnnc1, Mybpc1
Other molecular function	94	0.222994	Aox1, F13a1, ApoE, Pgc, Neb, Hnrnpa1, Mrfap1, Thbs1, Slc4a1, Lmnb1, Ngp, Fabp5, Hist1h1b, Dpm1, Siae, Vcan, Anxa1, Sbp, Gemin4, Tsn, Hist1h1d, Ptx3, A2m, Acy1, Mgp, Pgm1, Tnnc2, Apof, Tpm2, Car3, Ptgr1, Ldlr, Acan, Glrx3, Chi3l1, Nono, Car1, Mpo, Hpx, Fabp4, Mpst, Fn1, Abp1, Igfbp1, Fabp3, Hspa1l, Col1a2, Retnlg, Rps28, Pfkp, Lcn2, Chad, Tceb2, S100a11, Hba-a2, Ostf1, Fabp6, Fh1, Myh6, Sema6d, Pgam2, Lbp, Tpi1, Trim72, Pgls, Hspa5, Pla1a, Ldha, Ckm, Tsku, Hprt1, Ckb, Pdlim3, Ak1, Itpa, Serping1, Eno3, Tnnc1, Semg1, Gstm1, Hp, Col1a1, Rpl8, Mb, Atp7a, Orm1, Slpi, S100a8, Hexb, Col6a2, S100a9, Atp5a1, Limd2, Prg4

**Table VI:** Proteins in signaling pathway.

Title	Gene count	P-value	Genes
ECM-receptor interaction	7	5.98E-05	Thbs1,Col6a3,Fn1,Col1a2,Chad,Col1a1,Col6a2
Complement and coagulation cascades	6	0.000165	F13a1,Kng1,C4bpb,A2m,C4bpa,Serping1
Focal adhesion	7	0.010068	Thbs1,Col6a3,Fn1,Col1a2,Chad,Col1a1,Col6a2
Cardiac muscle contraction	5	0.002137	Tpm2,Tpm3,Myh6,Tpm1,Tnnc1
PPAR signaling pathway	4	0.009466	Fabp5,Fabp4,Fabp3,Fabp6
Renin-angiotensin system	1	0.187962	Agt
Ribosome	2	0.271977	Rps28,Rpl8
Antigen processing and presentation	2	0.290114	Hspa1l,Hspa5
Nucleotide excision repair	1	0.417476	Ddb1
Ubiquitin mediated proteolysis	2	0.484216	Ddb1,Tceb2
Calcium signaling pathway	2	0.643433	Tnnc2,Tnnc1
TGF-β signaling pathway	1	0.653817	Thbs1
Hematopoietic cell lineage	1	0.658099	Il1r2
Endocytosis	2	0.664494	Ldlr,Hspa1l
Toll-like receptor signaling pathway	1	0.712848	Lbp
Vascular smooth muscle contraction	1	0.755917	Cald1
Lysosome	1	0.758953	Hexb
Axon guidance	1	0.79776	Sema6d
Tight junction	1	0.805238	Myh6
Cell adhesion molecules	1	0.80767	Vcan
MAPK signaling pathway	2	0.848604	Hspa1l,Il1r2
Regulation of actin cytoskeleton	1	0.930221	Fn1
Cytokine-cytokine receptor interaction	1	0.96282	Il1r2
Olfactory transduction	1	0.993046	Clca1
